# The Roles of TNFR2 Signaling in Cancer Cells and the Tumor Microenvironment and the Potency of TNFR2 Targeted Therapy

**DOI:** 10.3390/cells11121952

**Published:** 2022-06-17

**Authors:** Hiroyuki Takahashi, Gumpei Yoshimatsu, Denise Louise Faustman

**Affiliations:** 1 Department of Gastroenterological Surgery, Fukuoka University Hospital, Fukuoka 814-0180, Japan; htakahashi3@mgh.harvard.edu (H.T.); gumpei.ysohimatsu@fukuokauniversityhospital.org (G.Y.); 2 Immunobiology Department, Massachusetts General Hospital and Harvard Medical School, Charlestown, MA 02129, USA

**Keywords:** TNFR2, tumor microenvironment, immune checkpoint inhibitor, Tregs, immunotherapy, anti-TNFR2 antibody

## Abstract

The appreciation that cancer growth is promoted by a dynamic tumor microenvironment (TME) has spawned novel approaches to cancer treatment. New therapies include agents that activate quiescent T effector cells and agents that interfere with abnormal neovascularity. Although promising, many experimental therapies targeted at the TME have systemic toxicity. Another approach is to target the TME with greater specificity by taking aim at the tumor necrosis factor receptor 2 (TNFR2) signaling pathway. TNFR2 is an attractive molecular target because it is rarely expressed in normal tissues (thus, has low potential for systemic toxicity) and because it is overexpressed on many types of cancer cells as well as on associated TME components, such as T regulatory cells (Tregs), tumor-associated macrophages, and other cells that facilitate tumor progression and spread. Novel therapies that block TNFR2 signaling show promise in cell culture studies, animal models, and human studies. Novel antibodies have been developed that expressly kill only rapidly proliferating cells expressing newly synthesized TNFR2 protein. This review traces the origins of our understanding of TNFR2’s multifaceted roles in the TME and discusses the therapeutic potential of agents designed to block TNFR2 as the cornerstone of a TME-specific strategy.

## 1. Introduction

Since the identification of nitrogen mustard as an anti-cancer drug in 1940s, pharmaceutical options for malignant disease have remarkably progressed to increasingly targeted therapies [1]. Cancer immunotherapy using immune checkpoint inhibitors (ICIs) such as anti-programmed cell death protein 1 (PD-1) drugs or anti-cytotoxic T lymphocytes-associated protein 4 (CTLA-4) drugs have become common in the clinical setting [2]. ICIs harness the human immune system to attack tumors, predominantly by enhancing the anti-tumor activity of cytotoxic T lymphocytes (i.e., effector T cells, Teffs) [3]. While ICIs may prolong survival, tumors often acquire primary or secondary resistance to them [4]. Further, ICIs have unavoidable adverse events because they act broadly on the immune system. Adverse events include autoimmune disease, cardiotoxicity, and cytokine release syndrome [5,6]. Recent attention has focused on targeting the tumor microenvironment (TME), which largely consists of infiltrating immune cells, notably T-regulatory cells (Tregs), neo-vascular cells, stromal cells, and tumor cells [3,7]. Interactions between cancer cells and other cells of the TME affect overall tumor growth, homeostasis, and progression [3]. Uncovering TME’s detailed roles in tumor progression has led to a shift in anti-tumor therapies to include TME’s supportive molecules, such as protein kinases, endothelial growth factors, and superficial receptors [8]. Drugs directed at these molecular targets are thought to exhibit less systemic toxicity vs. conventional chemotherapy but they still exert systemic effects [9,10]. Thus, the ideal anti-cancer strategy for the next era may require targeting molecules more specifically expressed in the TME.

One component of the TME is tumor necrosis factor (TNF) and its receptors. TNF is a unique cytokine that exerts two distinct actions depending on its two receptors; one is tumor necrosis factor receptor 1 (TNFR1), which initiates inflammation and tissue apoptosis and/or necrosis depending on the cell type affected, and the other is TNFR2, which modulates the immune system and tissue regeneration [7,11]. Although both receptors are part of the human TNF superfamily, they are linked to separate intracellular signaling pathways. The TNFR1 surface receptor is linked to a cell death pathway, while TNFR2 is linked to a cell proliferation pathway. Consequently, TNF-related signaling may exert both tumor promotion and suppression [12]. While TNFR1 has been widely studied with respect to the TME, TNFR2 has received scant attention. One possible reason is that TNFR1 is diffusely expressed on mammalian cells, so it was identified much earlier than TNFR2 [13]. Yet, as more knowledge emerges, it has become apparent that TNFR2 is overexpressed in many cancers and in other cells of the TME, while it is rarely expressed in normal tissue. Therefore, a greater understanding of this axis may yield clinical benefits for cancer patients. In this review, we outline recent developments in the pathophysiology of TNFR2 signaling in the TME and the benefits of targeted therapies that block TNFR2 signaling.

## 2. The Structure and Function of TNF/TNFR

**Figure 1:**
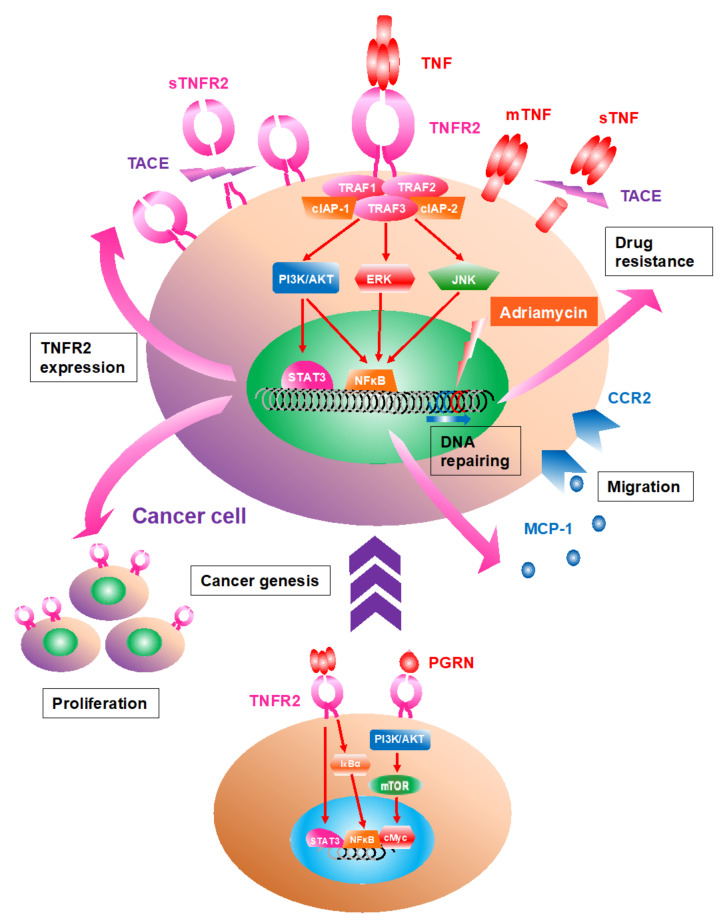
The roles of TNFR2 signaling within cancer cells. Many types of cancer cells express tumor necrosis factor receptor 2 (TNFR2) on their surface as well as trimeric membrane TNF, whose expression is also upregulated via TNFR2 signaling. After TNF binding, TRAF1/2/3 and clAP1/2 located on the intracellular domain of TNFR2 are clustered, activating further downstream pathways consisting of phosphatidyl inositol 3 kinase (PI3K)/AKT, mitogen-activated protein kinase (e.g., extracellular signal-regulated kinase, ERK; c-jun N-terminal kinase, JNK), nuclear factor-κ B (NFκB), and signal transducer and activator of transcription 3 (STAT3). The activated TNFR2 signal facilitates proliferation and migration of the cancer cell as well as autocrine signaling by macrophage chemotaxis protein-1 (MCP-1) via the C-C chemokine receptor type 2 (CCR2), which drives the cancer cell to invade or migrate. Malignant transformation and tumorigenesis are induced via the signal; TNF or progranulin (PGRN) binding to TNFR2 activates IκBα and NFκB or PI3K/AKT/mammalian target of rapamycin (mTOR) and cMyc. The colitis-associated carcinogenesis is also developed by NFκB activation. In addition, TNF and TNFR2 are cleaved in their stem regions by TNFα-converting enzyme (TACE), resulting in their soluble forms, sTNF and sTNFR2.

In 1975, Carswell and his colleagues initially identified TNF as a cytokine that leads to the necrosis of tumor tissue [14]. Indeed, TNF stands for tumor necrosis factor. However, TNF has become recognized as a multi-functional molecule that controls tissue homeostasis in various and paradoxical ways, including proinflammation, regeneration, tissue necrosis, and cell proliferation [15,16]. This 34 kDa cytokine can be both a cytokine and found in the serum. TNF is also a type II transmembrane trimeric protein and a representative member of the TNF superfamily (TNFSF), consisting of 19 ligands and 29 receptors in humans [17]. TNF is composed of trimetric molecules and works by being membrane-attached (mTNF) or a solvable form (sTNF). The latter is cleaved from the stalk region on cellular surfaces by the metalloprotease TNFα-converting enzyme (TACE) (Figure 1) [18]. Activated signaling through TNFR2 by mTNF binding is usually more effective than stimulation by sTNF, which can be triggered only by an intact trimetric ligand [17]. Additionally, while mTNF activates both TNFR1 and TNFR2, sTNF activates only TNFR1 because sTNF binding to TNFR2 lacks secondary clustering of initial trimetric TNF-TNFR2 complex [19]. Tight trimerization is required for downstream signaling. TNF is produced by various cells, including activated macrophages, Teffs, fibroblasts, and keratinocytes, while production is suppressed by some immunomodulatory cells, such as Tregs and mesenchymal stem cells [20,21]. Lymphotoxin alpha (LTα or TNFβ), another soluble ligand belonging to TNFSF, and progranulin (PGRN), may also interact with these two receptors [22,23].

TNF receptor super family (TNFRSF), a member of type 1 transmembrane receptors, has co-evolved together with TNFSF, acquiring diversity by genetic duplication or transposition [24]. The TNFRSF consists of the ectodomain, transmembrane domain, and intracellular domain, usually from either a parallel or antiparallel dimer [25]. While the parallel dimer exposes the ligand binding site that activates the downstream signal pathway, the antiparallel dimer buries the ligand binding site to block activation when stimulated by an antagonistic antibody [17].

TNFR1 (TNFRSF1A or CD120a) is classified as a death receptor group in the TNFRSF, which includes the death domain (DD) in their intracellular portion [17]. Activated TNFR1 signaling can aggregate DD and lead to recruitment of TNFR1-associated death domain protein, Fas-associated death domain, receptor-interacting protein and mitogen-activated protein kinase (MAPK) activating death domain. This results in the downstream caspase-8 activation and typically cell death [26]. Simultaneously, the activated signaling can induce the enlargement of cell death-independent proinflammatory pathways activating nuclear factor-κ B (NFκB) and MAPK [27]. Thus, TNF signaling via TNFR1 plays crucial roles in the cellular apoptotic signaling mechanism. Additionally, the TNFR1 receptor is expressed on all cells in both the human immune lymphoid cells and parenchymal cells.

It is important to distinguish TNFR1 from the TNFR2 receptor—although their names are very similar and they both can bind TNF, their functions are very different. TNFR2 (TNFRSF1B or CD120b) is a representative of the TNFR-associated factor (TRAF)-interacting receptor subgroup of the TNFRSF and is an inducible receptor and lymphoid-only expressing receptor. Additionally, TNFR2 is inducible unlike the near constant expression of TNFR1 on all mammalian cells [17]. Stimulation via mTNF can suppress inflammation by downregulating the NFκB pathway, resulting in cell survival and proliferation [17]. After TNF binding, TNFR2 recruits adaptor proteins such as TRAF and clAP, which translocate activated NFκB and c-Jun N-terminal kinase (JNK) into the nucleus [28,29]. In addition, when TNFR2 trimerizes by mTNF binding, this tightly clustered complex becomes susceptible to TACE and cleaved into the soluble form (sTNFR2) [30]. Soluble forms of TNFRSF usually work similar to the decoy receptor, which lack an intracellular domain and act as TNFSF ligand inhibitors [17]. Importantly, the evaluation of serum sTNFR2 level has been attractive in various cancer studies, owing to the long-term stability and sensitivity and is a sign of the marked agonism of this pathway [31].

The most determinative differences between TNFR1 and TNFR2 are: (1) the absence of a DD in TNFR2, (2) TNFR2’s linkage to the proliferative NFkB pathway, (3) ubiquitous expression of TNFR1 on all tissues, and (4) ineducability of TNFR2 at sites in inflammation and overall restricted cellular expression. Accordingly, we may roughly understand TNFR1-associated signaling as cellular death, while TNFR2-associated signaling as cellular proliferation/regeneration. Furthermore, the bodily distributions of these receptors are very different. While TNFR1 is normally expressed by almost all mammalian cells, TNFR2’s expression is limited to lymphocytes, myeloid, and endothelial cells [32]. The greater localization of TNFR2 expression is expected to be a therapeutic advantage in terms of fewer adverse events. Additionally, TNFR1 typically has constitutive expression; whereas TNFR2 expression is inducible by various cytokines. This also explains the differential expression and abuse of this receptor in the TME.

## 3. TNFR2 Expression Is Upregulated in Cancer Cells; TNFR2 Signaling Accelerates Proliferation

Human and murine TME features aberrantly high TNFR2 receptor expression (Table 1). The reason for overexpression of the TNFR2 protein for preferential cell growth is obvious but the cause(s) and mechanisms behind upregulation are diverse. For example, in human colorectal cancer, the overexpression of TNFR2 is driven via activated STAT3 through IL-6 and TNF induction (Figure 1) [33]. Activated TNFR2 signaling in malignant cells includes breast, lung, colorectal, renal, cervical, skin cancer, melanoma, and malignant lymphoma. The activation of phosphatidyl inositol 3 kinase (PI3K)/AKT, MAPK, STAT3, and NFκB promote the downstream signaling pathway in these cancer cells [22,23,33,34,35,36,37,38,39]. As another example, in vitro overexpression of TNFR2 in colorectal cancer cells increases their proliferation by 2-fold; conversely, knockdown of TNFR2 leads to significant downregulation of proliferation [33]. Similarly, TNFR2/p75 knockout mice in vivo exhibit more than 2-fold decrease in growth of Lewis lung carcinoma compared with wild type mice [40]. Thus, TNFR2 is overexpressed in a variety of cancers and its activation promotes tumor growth and progression.

## 4. Activated TNF/TNFR2 Signaling Is Associated with Proliferation and Recruitment of Stromal Cells in the TME

**Figure 2:**
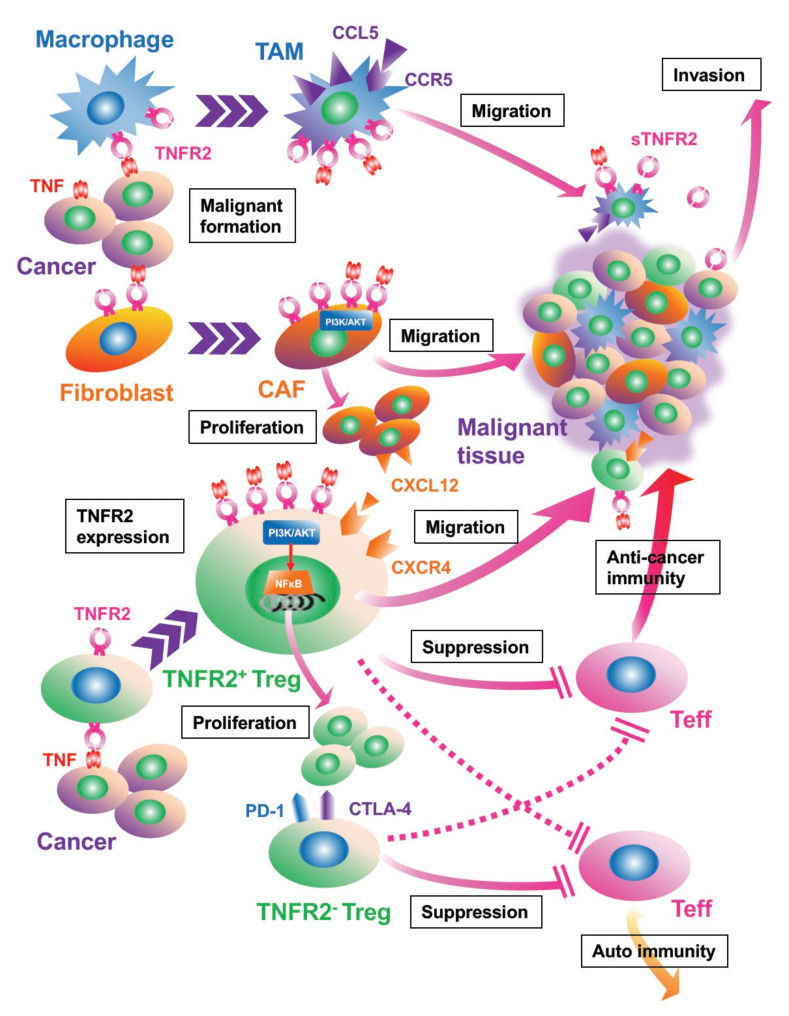
The interaction between cancer cells and stromal cells of the TME via TNFR2 signaling. TNFR2^+^ macrophages and fibroblasts are influenced by receptor binding of TNF, resulting in a transformation to tumor-associated macrophages (TAMs) and cancer-associated fibroblast (CAFs). These cells can accelerate tumor proliferation via phosphatidyl inositol 3 kinase (PI3K)/AKT activation and migration by autocrine or paracrine of chemokine C-C motif ligand 5 (CCL5)/CCR5. Malignant stromal cells are promoters of tumor burden and metastasis through angiogenesis and immunomodulation. In addition, sTNFR2 released from TAMs directly drives cancer invasion. TNFR2^+^ regulatory T cells (Tregs), which are a minor subpopulation in the normal state, accelerate proliferation and migration of malignant tissue via TNFR2 signaling and contribute to tumor growth by strongly suppressing effector T cells (Teffs). The degree of TNFR2 expression is associated with the suppressing function, expression of C-X-C motif chemokine receptor 4 (CXCR4), migration of Tregs, and predicts the prognosis of patients with malignant disease. Meanwhile, TNFR2^-^ Tregs, which usually express programmed cell death protein 1 (PD-1) and cytotoxic T lymphocytes-associated protein 4 (CTLA-4), are considered to play a critical role in immunomodulation in the normal state.

Stromal cells in the TME such as macrophages and fibroblasts can also express TNFR2 on their surface [56]. These cells undergo “malignant education”, which is a cellular activating process resulting in a switch from their normal into malignant phenotypes: tumor-associated macrophages (TAMs) and cancer-associated fibroblasts (CAFs), respectively (Figure 2) [49,57]. TAMs and CAFs are implicated in tumor progression by directly driving cancer cell proliferation and/or by enhancing angiogenesis and modulating immune cells [58]. Although the decoy receptor 3 is common as the TAMs promoter among the TNFRSF [59], TNFR2 signaling also seems to play an important role in the TME [49,60]. TAMs harvested from patients with triple negative breast cancer (TNBC) expressed higher TNFR2 as compared with those from non-TNBC patients [42]. Interestingly, the degree of TNFR2 expression in TAMs is strongly correlated with the chemokine C-C motif ligand 5 (CCL5) expression, which facilitates the recruitment of TAMs in the TME [42]. TNFR2 is dominantly expressed in CAFs as well, and the proliferation and migration can be accelerated through activated signaling [49,61]. Thus, these cancer-associated stromal cells are pivotal modulators of tumorigenesis.

## 5. Cancer Invasion and Metastasis Are Facilitated by TNFR2 Signaling

**Figure 3:**
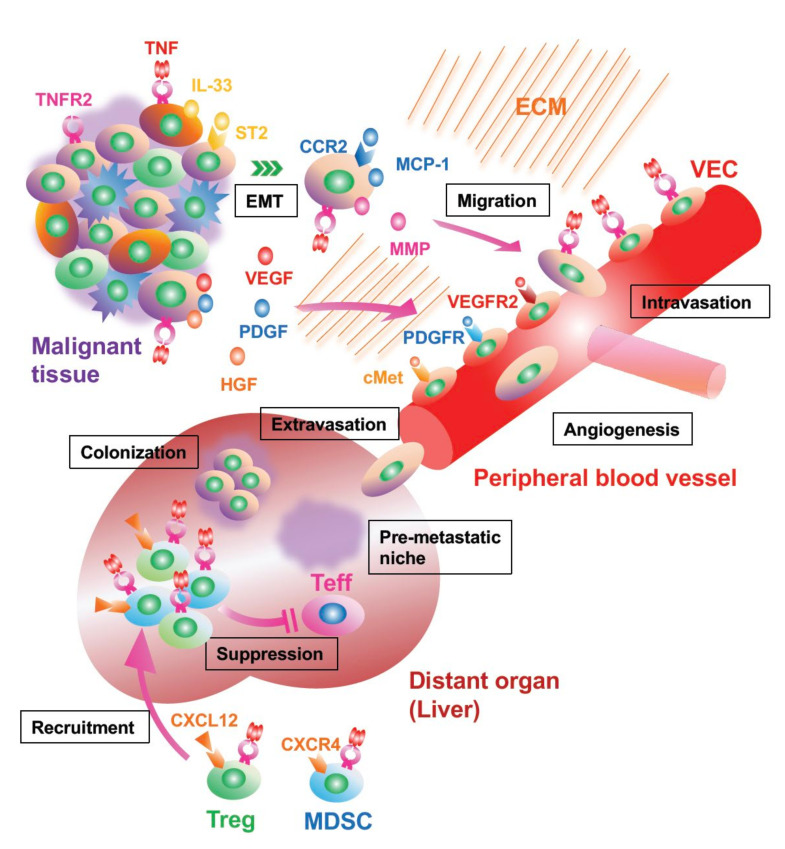
Emerging mechanisms of cancer metastasis via TNFR2 signaling. Cancer cells can leave malignant tissue and migrate into the extracellular matrix (ECM) by the endothelial mesenchymal transition (EMT), a process driven via IL-33 released from cancer-associated fibroblast (CAF) activated via TNF/TNFR2 signaling. By autocrine action of macrophage chemotaxis protein-1 (MCP-1) and secretion of matrix metalloprotease (MMP), the cancer cell invades into the blood capillary newly generated by angiogenesis. The proliferation of vascular endothelial cell (VEC) is also accelerated via TNFR2 signaling in addition to the interaction of vascular endothelial growth factor (VEGF)/VEGFR2, platelet derived growth factor (PDGF)/PDGFR, and hepatocyte growth factor (HGF)/cMet. Before and after the metastasis, TNFR2^+^ regulatory T cells (Tregs) and myeloid-derived suppressor cells (MDSCs) are recruited to the site from bone marrow with upregulated C-X-C motif chemokine receptor 4 (CXCR4) expression after the binding of TNF. These immunosuppressor cells can establish premetastatic niches by downregulating effector T cells (Teffs).

For widely metastatic cancers, TNFR2 may play a role in promoting the metastatic process and its progression. In metastatic disease, cancer cells from the primary lesion invade the local parenchymal tissue and then intravasate into either blood or lymphatic vessels to enter the circulation [62]. Following this dissemination step, the cancer cells are trapped in the microvessels at distant organs, where they extravasate from the circulation to enter the parenchymal tissue, resulting in the colonization at the site [62]. To date, some studies have shown the association of TNFR2 signaling with the metastatic process (Figure 3). In murine colon and lung cancer models, hepatic colonization and growth of the metastatic lesion are promoted via TNFR2-activated signaling [45]. Conversely, the induced genetic loss of TNFR2 in the mice decreases metastasis of melanoma within the lung [53].

There is support for activated TNFR2 signaling driving the invasion and migration of cancer cells. In multiple myeloma patients, for example, transendothelial migration through the bone marrow endothelial cell monolayer is accelerated by the autocrine action of monocyte chemoattract protein-1 (MCP-1) following activated TNFR2 signaling (Figure 2) [54]. In addition, sTNFR2 derived from TAMs directly promotes the invasiveness of TNBC [42].

The neogenesis of blood or lymphatic vessels in the TME can be facilitated by activated TNFR2 signaling. In renal cell carcinoma, greater TNFR2 expression on vascular endothelial cells (VECs) is observed with higher malignant grades [37]. The activated signaling promotes angiogenesis by upregulating vascular endothelial growth factor (VEGF), hepatocyte growth factor (HGF) and platelet derived growth factor (PDGF) [22,23,36,63]. Conversely, vascular regeneration is impaired in TNFR2^−/−^ mice [36]. The neovascularity may promote metastatic disease but also perhaps tumor nutrient resources [64]. TNFR2 downregulation in human papillary thyroid carcinoma occurs using H19 long non-coding RNAs [41]. H19 inhibits TNFR2 expression and may be used as a potential diagnostic tool and therapeutic target for this cancer.

TNFR2 signaling also may play a role in driving epithelial mesenchymal transformation (EMT) and releasing matrix metalloprotease (MMP) during the tumor infiltration process. EMT is an acquired mesenchymal phenotypic alteration and essential biological process for cancer cells to migrate or intravasate [65]. It is known that EMT is promoted via IL-1*β*, transforming growth factor *β*, and TNF*α* signaling [65,66]. Of great interest, CAFs-derived IL-33 can induce EMT of gastric cancer cells, which is facilitated via activated TNFR2 signaling [46]. On the other hand, MMP is an important tool for tumor cells to invade into the extracellular matrix by destroying connective tissue. TNF released by cancer cells or TAMs is related to MMP [67]. Tanimura and colleagues showed MMP secretion via activated TNFR2 signaling following cancer invasion with the cholangiocarcinoma cell line which displays only TNFR2 [48].

TNFR2 signaling exerts an important regulatory role in distant metastatic sites both before and after colonization. Ham and colleagues demonstrated that signal activation could recruit myeloid-derived suppressor cells (MDSCs) into the distant liver in colorectal cancer. This action suppresses anti-cancer immunity and stabilizes the colonization [45]. Curiously, the recruitment occurred even before the colonization of the metastatic cancer cells [45]. Several tumor-derived cytokines and molecules play a necessary role in mobilizing tumor-supportive host cells from the distant anatomical sites, known as the premetastatic niche [62]. These host cells are rendered pro-tumorigenic even prior to their mobilization, indicating that the MDSCs induced via TNFR2 signaling may contribute to the establishment of the premetastatic niche.

## 6. TNFR Signaling Controls Immunomodulating Cells, Tregs, and MDSCs

Limiting host anti-cancer immunity is important for malignant progression. The immunomodulating cells with this potential are also essential components of the TME. Both TME Tregs and MDSCs rely on the TNFR2 signaling pathway.

Increased numbers of Tregs are found in the TME, even in the peripheral tissue of cancer patients, and Tregs play a master role in the suppression of anti-tumor immunity (Figure 2) [50,68]. Owing to the highly suppressive potential of Tregs, Teffs are not potent enough for killing the tumor. TNFR2 signaling propels the differentiation of Tregs in the thymus, and their expansion and stabilization through epigenetic mechanisms [69,70,71]. Herein, significant TNFR2 expression is found in the tumor-infiltrating Tregs, and the activated TNFR2 signaling facilitates the proliferation and suppressive function via PI3K/AKT and/or NFκB activation in various cancers. Tregs are known to promote diverse cancers such as breast cancer, lung cancer, colorectal cancer, ovarian cancer, malignant lymphoma, melanoma, and leukemia [25,44,50,52,53,55,72,73,74,75]. Although Tregs expressing TNFR2 are only a subpopulation of total Tregs in the human TME, the degree of TNFR2 expression is tenfold higher than that of TNFR1 in human blood, and the TNFR2^+^ Tregs are equipped to be the most suppressive subset against Teffs [52,70,76]. In TNFR2-deficient mice, more Teff numbers are found, and thus, tumor control is potentiated [77]. In humans, TNFR2^+^ Tregs in acute myeloid leukemia (AML) patients also display higher levels of C-X-C chemokine receptor type 4 (CXCR4) expression, which is positively correlated with TNFR2 expression levels on Tregs [55]. This suggests that the CXCR4/CXCL12 axis may play a role in the accumulation of TNFR2^+^ Tregs in the TME. Although TNFR2 is expressed on Teffs as well, much higher levels are found in the Tregs of the TME [44,52].

Activated TNFR2 signaling controls the proliferation and suppressive activity of MDSCs [78]. MDSCs are often found in peripheral tissues and result in the tumor growth both at primary and distant metastatic sites [45,79]. A recent finding is that MDSCs accumulate and survive in the TME via activated TNFR2 signaling [80]. For instance, while mTNF-expressed cancer cells recruit these MDSCs into the TME, TNFR2 deficiency on MDSCs also impairs CXCR4 expression [81]. Thus, TNFR2 is dominantly expressed on these immunomodulatory cells in the TME.

Other immune cells such as dendritic cells and natural killer cells could be also affected by the TNF/TNFR2 signaling pathway [82]. However, it is still unclear how they work in TME; thus, future investigations are desired in this field.

## 7. Oncogenesis Can Be Driven by TNFR2 Signaling

Over the past decades, evidence of oncogenesis induced by TNFR2 signaling has accumulated. This should perhaps not be surprising since any receptor that preferentially promotes growth would directly favor oncogenesis. Initially, Onizawa and his group revealed that specific upregulation of TNFR2 was observed in inflamed intestinal epithelial cells and that TNFR2-mediated epithelial NFκB activation correlated with colitis-associated carcinogenesis (Figure 1) [83]. Additionally, the investigators extended their observation by demonstrating that the myosin light chain kinase expression was induced via TNFR2-signaling-mediated tumorigenesis [84].

Upregulated TNFR2 signaling in colonic epithelial cells leads to colitis-associated carcinogenesis [83]. TNFR2-induced activation of transcription factor-associated protein induced the malignant transformation of hepatic progenitor cells and liver tumorigenesis, and the recurrence of hepatocellular carcinoma via STAT3 activation [47,85]. Other studies reveal another mechanism using murine brain cancer models: activated mammalian target of rapamycin (mTOR) signaling pathway followed by PGRN binding to TNFR2 was identified in malignant cervical cancer cells, whereas the inhibition of mTOR suppressed malignant transformation and tumor growth [38]. He and colleagues reported the tumorigenesis by the loss of TNFR2 allele using a murine breast cancer model [86]. These studies suggest that the TNFR signaling pathway may promote oncogenesis.

## 8. TNFR2 Signaling in Cancer Stem Cells: Still Controversial

Cancer stem cells (CSCs) seed many if not most tumors, reigning on the top of the cancer hierarchy [87]. CSCs also can play a role in anti-cancer drug resistance [88]. Some studies suggest the TNFR2 signaling pathway is associated with stem cell proliferation, survival, and differentiation in non-malignant tissues [89,90].

TNFR2 signaling may negatively modulate CSCs in acute myeloid leukemia. Some research reveals that TNFR2 gene expression was significantly decreased in the leukemia samples and correlated with the inactivity of CSCs as compared with those from the non-leukemia control samples [91]. Conversely, Al-Lamki and colleagues showed that the proliferation of CD133^+^ CSCs in renal clear cell carcinoma was promoted by TNFR2 signaling [51,92]. Importantly, while most types of CSCs strictly limit the self-proliferation and bear the resistance to various chemotherapies, activated CD133^+^ CSCs via TNFR2 signaling could drive cellular division and sensitivity to chemotherapy drugs such as cyclophosphamide [51]. The relationship between the signaling and CSCs is only beginning to emerge.

## 9. TNFR2 Signaling Can Contribute to Chemotherapy Resistance

Some investigators have proposed that chemotherapy drug resistance is associated with TNFR2 signaling by cancer cells. The initial report was a negative correlation. Zhang and colleagues reported that apoptotic response of colorectal cancer cells to 5-fluorouracil was negatively mediated by induced TNFR2 [93]. In contrast, later reports indicated a positive association with TNFR2 signaling. Sprowl and coworkers showed that TNFR2 expression was significantly upregulated in Adriamycin-resistant human breast cancer cells [94]. In addition, Yang and colleagues also demonstrated the underlying mechanism by repairing DNA damage in cancer cells via an activated TNFR2/AKT pathway [43].

In metastatic melanoma, accumulating evidence suggests a TNFR2 escape mechanism after failed checkpoint inhibitor therapy. A detailed single cell mRNA sequencing study for both failed CTLA-4 and PD1 human cancer identified signature mRNAs indicative of Tregs (Foxp3) with upregulation. Additionally, this study found upregulated TNFR2, also known as the TNFRSF1B gene [95]. Similar data confirmed this is the setting of anti-PD1 therapy with pembrolizumab, even after one cycle of therapy. Tregs were abundant in the TME of humans with metastatic melanoma and retained their immunosuppressive phenotype and functionality following anti-PD-1 [96]. Epigenetic, transcriptomic, and proteomic analysis of Tregs after immune checkpoint therapies implicated TNFR2 signaling as a possible driver of CD8^+^ T cell suppression. TNFR2 was preferentially expressed by Tregs in the TME of humans with advanced disease. In parallel mouse studies, dual blockade of TNFR2 and PD-1 led to potent CD8^+^ T cell expansion in two mouse tumor models, and restored sensitivity to immune checkpoint inhibitors [97]. This was buttressed by a study showing that TNFR2 antagonistic antibodies administered to mice prior to checkpoint inhibitors led to effective Treg depletion and enhanced tumor survival rates in two models of colon cancer [98].

## 10. Anti-Cancer Therapy Targeting TNFR2; TNFR2^+^ Treg Depletion Leads to Beneficial Outcomes

Anti-TNFR2-based therapy holds promise for cancer treatment. TNFR2 is an attractive target because it is directly upregulated as an oncoprotein on cancer cells and associated components including TAMs, CAFs, Tregs, and MDSCs that facilitate tumor dissemination [25,99]. While several drugs modulating TNFR signaling are available for a few autoimmune diseases, TNFR2-specific targeted therapies are still challenging in the field of oncology [25]. However, reducing TNFR2 expression in malignant disease seems to show promise in the clinical setting, according to two recent trials. One clinical trial targeting TNFR2^+^ Tregs was performed on AML patients who were ineligible for intensive chemotherapy. Fourteen patients received combined therapy with azacytidine, the DNA methyltransferase inhibitor, and panobinostat, the histone deacetylase inhibitor [55]. The patients, prior to treatment, showed a significantly higher number of TNFR2^+^ Tregs, which also possessed high migration potential as compared with those from healthy donors [55]. After treatment, patients showed a significantly decreased number of TNFR2^+^ Tregs both in peripheral blood and bone marrow, while TNFR2^-^ Tregs were not lowered. A reduction in TNFR2^+^ Tregs was accompanied by increased interferon-*γ* and IL-2 released from Teffs in the bone marrow, and prolonged disease free survival [55]. In another clinical trial, the same group reported that combined therapy with azacytidine and lenalidomide, the immunomodulatory drug, also contributed to a reduced number of TNFR2^+^ Tregs and TNFR2 expression on T cells, resulting in the durable clinical remission in AML patients [100]. Curiously, this study found that lenalidomide can independently reduce TNFR2^+^ Tregs, which was enhanced by adding azacytidine in vitro [100]. The epigenetics-modulating drugs have already been tried in myelodysplastic syndrome and multiple myeloma treatment; however, the adverse effects, such as a bone marrow suppression, limit therapy [101].

The expression of TNFR2 on Tregs is, thus, a mechanism for hampered cancer elimination. Many early mouse and human studies confirmed Tregs with this protein marker as the most functionally potent subtype of Treg cells [52,76,102]. It was in cutaneous T cell lymphoma that data started to accumulate that TNFR2 could also act as an oncogene and be directly expressed on tumor cells for preferential growth [103].

## 11. Clinical Evaluation of TNFR2: Higher Levels of TNFR2 Expression Reflect Poor Prognosis and Risk of Cancer Development

Abundant and aberrant expression of TNFR2 on cancer cells and Tregs of the TME has led to the concept that this receptor could be used for diagnosis, prognosis, and deciding a treatment plan. Human studies have focused on sTNFR2 in peripheral blood, in TNFR2 in cancer cells and Tregs, and in metastatic tissue (Table 2).

Two overarching points emerge from Table 2. First, the degree of TNFR2 expression in malignant cells is positively associated with disease progression and prognosis. Higher TNFR2 expression is correlated with the highest risk of tumor growth in ovarian, esophageal, and breast cancer; moreover, TNFR2 gene expression in breast cancer cells also indicates a higher grade of malignancy [42,106,107,109,113]. Although higher levels of a protein are hard to quantify, two studies—one in human lung cell carcinoma and one in cutaneous T cell lymphoma—show this overexpression was actually logs of overexpression as measured by flow cytometry, an impressive signal [74]. Similarly, the number of TNFR2^+^ Tregs in malignant tissue indicates tumor progression. Compared to other commonly studied tumor markers such as OX40 and 4-1BB, TNFR2 overexpression was still 10× greater. The increased TNFR2 expression on Tregs is associated with lymphatic invasion, distant metastasis, and more advanced clinical stage in lung cancer. Furthermore, the increased degree of the TNFR2^+^ T cell populations among CD4^+^ T cells reflects relapse in AML [55,75,100].

Second, measuring sTNFR2 level in the serum is also useful. When there is augmented TNFR2 agonism, the receptor is shed. Elevated levels of sTNFR2 are a surrogate for extreme TNFR2 agonism [69]. For example, higher levels of sTNFR2 imply poor prognosis in colorectal, cervical cancer, and non-Hodgkin lymphoma [111,112,114,117]. A stage-dependent release of sTNFR2 into the bloodstream occurs in the endometrial cancer and an elevated sTNFR2 level indicates recurrence in glioblastoma [104,115]. Meanwhile, it reflects the therapeutic effect of radiotherapy in the head and neck cancer [105]. Importantly, the higher circulating sTNFR2 level can be a risk factor for cancer generation. Prude and his group analyzed the baseline serum levels of 67 immune and inflammation markers from 301 patients with non-Hodgkin lymphoma diagnosed over 5 years after the blood collection and 301 control patients within another disease screening trial, revealing that the higher sTNFR2 levels were strongly associated with lymphoma risk [118]. The relationship between elevated sTNFR2 level and cancer development also has been reported in endometrial and pancreatic cancer [110,116]. Thus, the serum sTNFR2 level serves as a novel biomarker that reflects TNFR2 activity, further the state of malignant disease, and also the drive of severe agonism of this receptor for preferential tumor growth.

## 12. Discussion

Specifically targeting TNFR2 is believed to be an ideal cancer therapy because TNFR2 expression is limited to minor subpopulations of normal cells, while its expression is enriched in the TME, including Tregs. The creation of TNFR2 antibodies with the ability to kill rapidly proliferating cells presents a rare example of a TME-specific cancer therapy [73,119]. Therefore, the targeting of TNFR2^+^ malignant cells with TNFR2-specific antagonistic antibodies may not only control cancer growth but also minimize adverse effects [25]. Here, we describe the current challenges to creating such potential drugs and discuss future perspectives on targeting TNFR2 therapy for malignant diseases. This discussion is based on Table 3, which is a summary of cell culture studies, animal models, and human studies of TNFR2-targeted antibody therapy for malignant diseases.

Early development of antibodies against TNFR2 was fraught with limitations. The antibodies worked in culture but did not work well in animal models. This was in large part driven by the fact that in vitro they blocked apparent signaling, but in vivo a tiny bit of TNF superfamily ligand uniformly caused their failure. After nearly 15 years of research, a novel type of true antagonistic antibodies was created [73,119]. Newly synthesized TNFR2 on rapidly proliferating cells was frozen as TNFR2 receptors into an anti-parallel dimer, thus, blocking the TNF binding site. Additionally, these antibodies only killed rapidly proliferating cells and left alone preformed TNFR2 timers, suggesting a more favorable structural biology to prevent off-target effects on circulating non-proliferating Tregs [73]. These antibodies have been called “dominant” antagonistic antibodies due to their ability to diminish human ovarian cancer cells in the ascites and cutaneous T cell lymphoma, even under TNF induction. The other type of antibody is called “recessive,” because it cannot overcome a tumor burden with the additional TNF [73,74]. The dominant antibody also inhibits Treg proliferation, especially strongly suppressive Tregs, in the TME only, resulting in activated Teffs [25]. There are some mechanistic clues supporting their usefulness:

(1) The dominant antibody targets a different site on the TNFR2 protein from the recessive antibody, resulting in stabilized TNFR2 in an antiparallel dimer formation and hexagonal lattice on the cellular surface, which blocks the activation of the signaling pathway [17,73].
(2) The dominant antibody does not require Fcγ receptor-mediated antibody binding [18,73].
(3) The dominant antibody inhibits the cleavage of the receptor from the cellular surface [73].
(4) We also expect that this dominant anti-TNFR2 antibody may overcome the drug resistance of CSCs with the expectation of achieving a complete cure in some malignant diseases.

For example, renal CSCs are not sensitive to chemotherapy until they proliferate through activated TNF/TNFR2 signaling [51]. This indicates that the dominant anti-TNFR2 antibody may effectively remove such drug-resistant CSCs.

Anti-TNFR2 antibody dominance may optimize therapy in additional ways. Using the chimeric mutant constructs and a structural biological technique, the IgG2 isoform of the antibody effectively kills rapidly growing cancer cells and immunosuppressive Tregs with hyperexpressed TNFR2 [119]. This accomplishment demonstrates that the stabilization of the antibody’s hinge and expansion of its arms may achieve the greatest efficacy as a cancer immunotherapy [17]. Furthermore, our anti-TNFR2 antibody, when combined with an ICI, effectively eliminates murine colorectal cancer [98]. Mice treated with the combination therapy displayed significantly decreased Treg and increased Teff number in their TME [98]. Importantly, the anti-TNFR2 antibody alone also displayed significantly longer survival in the treated mice as compared with the non-treated mice, suggesting that the antagonist may be an alternative Treg inhibitor for patients with tolerance to the existing ICIs.

Other research groups have attempted to identify specific anti-TNFR2 antibodies for cancer treatment. Nie and the coworkers combined their antagonistic anti-TNFR2 antibody, TR75-54.7, with an anti-CD25 antibody as therapy in murine colorectal and breast cancer models; they found significantly fewer TNFR2^+^ Tregs and more infiltrating IFN-*γ*^+^ Teffs, and remarkably decreased tumors [72]. The rationale for the combined therapy was to achieve synergy between the anti-TNFR2 antibody and an immune stimulant. More recently, Tam and colleagues developed another anti-TNFR2 antibody, Y2, which binds to the receptor outside of the TNF-binding site and accelerates the anti-tumor activity as Fc-dependent agonism of conventional T cells [120]. While Y2 facilitated the expansion and function of Teffs and downregulated the TNFR2 expression on T cells, Tregs were not depleted. Spontaneous immune cell activation never occurred in the healthy or tumor-bearing mice [120]. These results indicate less toxicity in the cancer therapy using the anti-TNFR2 antibody. The reason of less autoimmune effects by targeting TNFR2 therapy relative to ICIs may be intelligible by our proposed mechanism. Tregs expressing PD-1 and CTLA-4 act as critical immunomodulators, hence, severe side effects can be expected when targeting these immune checkpoint molecules. Meanwhile, TNFR2^+^ Tregs belong to the minor subpopulation of potent Tregs in the normal condition but can highly infiltrate in TME (Figure 2) [25].

As described above, a universal demethylation inhibitory drug such as azacytidine contributes to improved outcomes in some blood cancers by downregulating TNFR2^+^ Tregs [55]. Thus, drugs in this class, which regulate by an epigenetic mechanism, may be a potential treatment for malignant disease. However, to our knowledge, no studies have thus far directly investigated DNA methylation/demethylation of TNFR2 in malignant disease; it has not been uncovered whether the gene duplication and/or activating mutations occur in the malignant tissue, and it is not known whether gene duplication and/or activation occurs in the malignant tissue [121,122]. The epigenetic axis has shed light on the current cancer research and the detailed mechanism including PD-1 and CTLA-4 [121].

In conclusion, the TNFR2 signaling pathway plays pivotal roles in cancer progression. Targeted anti-TNFR2 therapy is known to deplete malignant cells and immunosuppressive Tregs, conferring both tumoricidal capacity. The evidence base developed thus far suggests that that more detailed investigations of TNFR2 signaling in the TME are desirable and that specific targeting of TNFR2 in the TME is an exciting new strategy for cancer immunotherapy.

## Figures and Tables

**Table 1:** Summary of TNFR2 Signaling-Related Mechanisms in the Tumor Microenvironment.

Malignant Disease	Human (C, Cancer Cell Line; P, Primary Cancer Cell)	Animal (M, Mouse; R, Rat)
Thyroid	(P) Non-coding RNA H19 inhibits lymphatic metastasis via TNFR2 downregulation [41].	
Breast	(P) TNFR2 is more highly expressed on TAMs [42]. (C) TNFR2 signaling contributes to Adriamycin resistance by repairing DNA damage via AKT signaling [43].	(M) TNFR2 signaling promotes cancer cell proliferation via activated p42/p44 MAPK [34]. (M) TNFR2^+^ Tregs have greater suppressive function over TNFR2^+^ Teffs [44].
Lung		(M) Tumor growth and angiogenesis are driven via TNFR2 signaling [36]. (M) TNFR2 signaling facilitates MDSCs-mediated immunosuppression and metastasis [45]. (M) TNFR2^+^ Tregs have greater suppressive function, which overcomes TNFR2^+^ Teffs [44].
Stomach	(P) CAFs-derived IL-33 released via TNF/TNFR2 signaling promotes gastric cancer metastasis [46].	
Liver		(R) TNFR2 signaling promotes recurrence of HCC via STAT3 activation [47].
Gallbladder	(C) MMP secretion and increased invasiveness are driven via TNF/TNFR2 signaling [48].	
Colorectal	(C) IL-6 and TNF induce TNFR2 expression via STAT3 activation [33]. (C) TNFR2 signaling promotes tumor growth via PI3K/AKT pathway [35]. (C) PGRN promotes cancer cell proliferation and angiogenesis via TNFR2/AKT pathway [23]. (C) PGRN/TNFR2 drives proliferation and migration of CAFs via AKT or ERK pathway [49].	(M) TNFR2 signaling facilitates MDSC-mediated immunosuppression and liver metastasis [45]. (M) TNFR2 signaling enhances Treg’s suppressive function [50].
Kidney	(P) and (C) TNFR2 signaling increases tumor progression via Etk-VEGFR2 cross talk [37]. (P) TNFR2 signaling drives CSC’s proliferation and increases their sensitivity to cytotoxicity [51].	
Ovary	(P) TNFR2^+^ Tregs in malignant ascites are more suppressive than those in blood [52].	
Uterus	(C) PGRN/TNFR2 signaling is needed for malignant transformation via mTOR signaling [38].	
Skin/Lymphoma	(C) LTα/TNFR2 signaling promotes tumor growth and angiogenesis in cutaneous lymphoma [22].	(M) Both TNFR1 and TNFR2 are necessary for optimal TNF signaling during skin cancer development [39].
Melanoma		(M) Tumor growth and angiogenesis are driven via TNFR2 signaling [36]. (M) TNFR2 signaling expands Tregs, driving lung metastasis [53].
Leukemia/Myeloma	(C) Transendothelial migration is driven via TNFR2 signaling and upregulated MCP-1 secretion [54]. (P) Compared to TNFR^-^ Tregs, TNFR2^+^ Tregs express higher CXCL4, which facilitates migration in AML [55].	

TNFR2, tumor necrosis factor receptor 2; TAMs, tumor-associated macrophages; CAFs, cancer-associated fibroblasts; EMT, endothelial mesenchymal transition; MMP, matrix metalloprotease; STAT3, signal transducer and activator of transcription 3; PI3K, phosphatidyl inositol 3 kinase; PGRN, progranulin; ERK, extracellular signal-regulated kinase; VEGFR2, vascular endothelial growth factor 2; CSCs, cancer stem cells; Tregs, regulatory T cells; mTOR, mammalian target of rapamycin; LTα, lymphotoxin α; MCP-1, macrophage chemotaxis protein-1; CXCL4, C-X-C chemokine receptor type 4; AML, acute myeloid leukemia; MAPK, mitogen-activated protein kinase; Teffs, effector T cells; MDSCs, myeloid-derived suppressor cells; HCC, hepatocellular carcinoma.

**Table 2:** Summary of Human Studies of TNFR2 in Malignant Diseases.

Malignant Disease	Peripheral Blood	Primary Region	Metastatic Region
Brain	Plasma sTNFR2 levels are significantly higher in patients with recurrent GBM than healthy donors [104].		
Head and Neck	Decreased plasma sTNFR2 levels reflect treatment efficacy at 3 months post-IMRT [105].		
Breast		TNFR2 expression is positively correlated with increased tumor size, advanced clinical stage, poor differentiation, shorter OS, DFS [106], and tumor growth and angiogenesis [107].	Increased TNFR2^+^ B cells in metastatic LN are related to good prognosis [108].
		TNFR2 expression on TAMs is upregulated in TNBC compared to non-TNBC [42].	
Lung	Tregs express higher levels of TNFR2 than Teffs, and higher TNFR2^+^ Tregs are related to poor prognosis [75].		
Esophagus		TNFR2 expression is positively related to an advanced clinical stage, poor differentiation, and poor OS [109].	
Pancreas	Higher plasma sTNFR2 levels are marginally associated with a higher risk of pancreatic cancer [110].		
Colorectal	A higher plasma TNFR2 level is related to shorter OS in patients with metastasis after second-line chemotherapy [111]. Pre-diagnosis higher plasma sTNFR2 levels are associated with shorter OS [112].		
Ovary		TNFR2 expression is higher in cancer cells than in benign ovarian cells, and the elevated expression reflects cancer progression [113].	TNFR2^+^ Tregs are abundantly present in malignant ascites and show more suppressive characteristics than those in the peripheral blood [52].
Uterus	Peripheral TNFR2^+^ Tregs and circulating sTNFR2 are increased, and the percentage of TNFR2^+^ Tregs is inversely correlated with clinical stage [114]. Higher plasma sTNFR2 levels are associated with advanced clinical stage [115] and cancer risk [116].	Tumor-infiltrating TNFR2^+^ Tregs are significantly increased [114].	
Leukemia/Melanoma		Both total and TNFR2^+^ Treg populations are significantly higher in AML patients as compared with healthy donors [55].	
		TNFR2^+^ Tregs have higher levels of CTLA-4, Ki67 and CXCR4 as compared with TNFR2^-^ Tregs in AML patients [55].	
		Higher TNFR2^+^ Tregs and lower TNFR2^+^ Teffs are observed in AML patients as compared with healthy donors, and increased TNFR2^+^ Tregs are related to cancer relapse [100].	
Lymphoma	Higher plasma sTNFR2 levels are related to higher NHL risk [117,118].		

sTNFR2, soluble tissue necrosis factor receptor 2; GBM, glioblastoma; IMRT, intensity-modulated radiation therapy; Tregs, suppressive T cells; Teffs, effector T cells; OS, overall survival; NHL, non-Hodgkin lymphoma; DSF, disease free survival; TAMs, tumor-associated macrophage; TNBC, triple negative breast cancer; CTAL-4, anti-cytotoxic T lymphocytes-associated protein 4; CXCR4, C-X-C chemokine receptor type 4; AML, acute myeloid leukemia; LN, lymph node.

**Table 3:** Summary of studies using TNFR2-targeted therapy for malignant diseases.

Author (Year) [Ref.]	Drug	Specimen/Tissue	Mechanism/Outcomes
Govindaraj, C. et al. (2014) [55]	Combination of azacytidine and panobinostat	NFR2^+^ Tregs in AML patients	The combination therapy might inhibit DNA methyltransferase 1 and histone deacetylase in AML patients. The level of TNFR2^+^ Tregs in peripheral blood and bone marrow of patients was decreased, while the population of TNFR2^-^ Tregs was not reduced. Positive clinical response corresponded to decreased TNFR2^+^ Tregs and increased IFN-*γ*^+^ Teffs in bone marrow before vs. after treatment.
Govindaraj, C. et al. (2014) [100]	Combination of azacytidine and lenalidomide	NFR2^+^ Tregs in clinically remitted AML patients	Lenalidomide reduced TNFR2^+^ Tregs and augmented Teffs, which was enhanced by azacytidine, resulting in prolonged clinical remission.
Torrey, H. et al. (2017) [73]	Anti-TNFR2 antibody	Human Tregs in ovarian cancer ascites Human ovarian cancer cell line	The antibody bound to identical TNFR2 regions and locks in receptor resting state as antiparallel dimer form, which blocks downstream signaling pathway. Proliferation of Tregs was inhibited, while Teffs were expanded. In addition to TNFR2^+^ Tregs, TNFR2^+^ cancer cells were directly killed.
Nie, Y. et al. (2018) [72]	Combination of anti-TNFR2 antibody and anti-CD25 antibody	TNFR2^+^ Tregs in murine colorectal and breast cancer model	Remarkably decreased TNFR2^+^ Tregs and increased IFN-*γ*^+^ Teffs infiltrated in the tumor. Long term tumor-free survival was improved in murine cancer models.
Torrey, H. et al. (2019) [74]	Anti-TNFR2 antibody	TNFR2^+^ cancer cells and Tregs from patients with stage IV SS	TNFR2^+^ SS tumor cells and Tregs were dose-dependently decreased by the antibody, while beneficial and rapid expansion of Teffs was observed.
Tam, E. M. et al. (2019) [120]	Anti-TNFR2 antibody	Murine colorectal, breast, fibroblast fibrosarcoma, and B cell lymphoma cell lines	The antibody bound to outside of the TNF-binding region in TNFR2 and showed anti-tumor activity as Fc-dependent agonism of Teffs. Anti-TNFR2 treatment is mediated by Teffs and NK cells, downregulates TNFR2 on T cells, which leads to Teffs expansion and improved functionality. The antibody did not deplete Tregs and never caused spontaneous immune cell activation.
		Murine TNFR2^+^ Tregs	
Yang, M. et al. (2020) [119]	Anti-TNFR2 antibody	Human colorectal cancer, lymphoma and leukemia cell line	The anti-TNFR2 antibody showed specific killing of TNFR2-expressing tumor cells and Tregs, but sparing Teffs, which proliferated. The IgG2 isotype of the antagonists functioned better than the IgG1 isotype. The mutations to its amino acid sequence stabilized the natural variability of the IgG2 isotype’s hinge and improved function.
		Human Tregs and Teffs in the blood	
Case, K. et al. (2020) [98]	Combination of anti-TNFR2 antibody and anti-PD-1 antibody	Murine colorectal cancer	The combination therapy had the greatest efficacy by complete tumor regression and elimination, and the next most effective therapy was anti-TNFR2 alone, whereas the least effective was anti-PD-1 alone. The mode of action was by killing Tregs and increasing Teffs.

TNFR2, tissue necrosis factor receptor 2; AML, acute myeloid leukemia; SS, Sézary syndrome; Tregs, regulatory T cells; IFN-*γ*, interferon-gamma; Teffs, effector T cells; NK cells, natural killer cells.

## Data Availability

Not applicable.
